# Innovative CDR grafting and computational methods for PD-1 specific nanobody design

**DOI:** 10.3389/fbinf.2024.1488331

**Published:** 2025-01-17

**Authors:** Jagadeeswara Reddy Devasani, Girijasankar Guntuku, Nalini Panatula, Murali Krishna Kumar Muthyala, Mary Sulakshana Palla, Teruna J. Siahaan

**Affiliations:** ^1^ Pharmaceutical Biotechnology Division, A.U. College of Pharmaceutical Sciences, Andhra University, Visakhapatnam, Andhra Pradesh, India; ^2^ Pharmaceutical Chemistry Division, A.U. College of Pharmaceutical Sciences, Andhra University, Visakhapatnam, Andhra Pradesh, India; ^3^ GITAM School of Pharmacy, GITAM Deemed to be University, Visakhapatnam, Andhra Pradesh, India; ^4^ Department of Pharmaceutical Chemistry, School of Pharmacy, The University of Kansas, Lawrence, KS, United States

**Keywords:** cancer immunotherapy, nanobody, programmed cell death protein-1, complementarity-determining region, Western blot, ELISA, dot blot

## Abstract

**Introduction:**

The development of nanobodies targeting Programmed Cell Death Protein-1 (PD-1) offers a promising approach in cancer immunotherapy. This study aims to design and characterize a PD-1-specific nanobody using an integrated computational and experimental approach.

**Methods:**

An *in silico* design strategy was employed, involving Complementarity-Determining Region (CDR) grafting to construct the nanobody sequence. The three-dimensional structure of the nanobody was predicted using AlphaFold2, and molecular docking simulations via ClusPro were conducted to evaluate binding interactions with PD-1. Physicochemical properties, including stability and solubility, were analyzed using web-based tools, while molecular dynamics (MD) simulations assessed stability under physiological conditions. The nanobody was produced and purified using Ni-NTA chromatography, and experimental validation was performed through Western blotting, ELISA, and dot blot analysis.

**Results:**

Computational findings demonstrated favorable binding interactions, stability, and physicochemical properties of the nanobody. Experimental results confirmed the nanobody’s specific binding affinity to PD-1, with ELISA and dot blot analyses providing evidence of robust interaction.

**Discussion:**

This study highlights the potential of combining computational and experimental approaches for engineering nanobodies. The engineered PD-1 nanobody exhibits promising characteristics, making it a strong candidate for further testing in cancer immunotherapy applications.

## 1 Introduction

Cancer immunotherapy marks a significant breakthrough in oncology by harnessing the body’s immune system to recognize and eliminate cancer cells (Sharma and Allison, 2015; Farkona et al., 2016). Immune checkpoint proteins like PD-1, PD-L1, and CTLA-4 are key regulators that dampen immune activity, allowing tumors to escape immune surveillance. Therapeutic inhibitors of these checkpoints can reinvigorate the immune system to attack malignancies, significantly boosting anti-tumor responses (Shiravand et al., 2022; Alturki, 2023; Stirling et al., 2022; de Miguel and Calvo, 2020). Among these, PD-1 has appeared as a pivotal target in immunotherapy. Located on T cells, PD-1 typically prevents autoimmunity by moderating immune responses (Fife and Pauken, 2011; Kuchroo et al., 2021). Nonetheless, various cancers exploit PD-1 to avoid immune detection. Blocking PD-1 with specific therapies can restore immune function against cancer, offering a promising approach to cancer treatment (Liu J. et al., 2021; Yi et al., 2022; Li et al., 2018).

Traditionally, monoclonal antibodies (mAbs) have been employed to target PD-1, but these large proteins come with limitations such as high production costs, complex manufacturing processes, and potential immunogenicity (Michot et al., 2016). In contrast, nanobodies, or single-domain antibodies, represent a novel class of antibody-derived therapeutics that offer several advantages over conventional mAbs. Nanobodies originate from the distinctive heavy-chain-only antibodies in camelids and consist exclusively of the variable domain. These small, single-domain proteins retain the antigen-binding capability of conventional antibodies but with improved properties, including higher stability, better tissue penetration, and lower immunogenicity (Zhang Y. et al., 2023; Yu et al., 2021; Bannas et al., 2017; Jin et al., 2023).

The usage of nanobodies in cancer immunotherapy holds significant promise because of their efficiency in precisely targeting tumors (Liu M. et al., 2021), combined with their ease of production and modification (Van Audenhove and Gettemans, 2016). Despite their potential, the design and development of effective nanobodies require precise structural and functional optimization. In silico techniques have become invaluable in this regard, enabling the rapid design, prediction, and assessment of nanobody candidates (Hashemi et al., 2021).

This study aims to design, produce, and experimentally validate nanobodies targeting PD-1, combining advanced computational tools with laboratory techniques to ensure specificity, stability, and binding efficacy. Using Complementarity-Determining Region (CDR) grafting, we engineered a nanobody with high affinity and specificity for PD-1. Structural prediction was conducted using AlphaFold2, followed by molecular dynamics (MD) simulations to assess the stability and conformational dynamics of the nanobody under physiological conditions. Physicochemical properties such as stability and solubility were evaluated using various web-based tools to ensure optimal functionality. Molecular docking simulations further corroborated the interaction between the nanobody and PD-1, reinforcing its potential for applications in cancer immunotherapy. To validate the computational findings, we produced the nanobody using an expression system, followed by purification with Ni-NTA chromatography. Experimental characterization was performed using Western blotting and ELISA to nanobody binding affinity to PD-1, and dot blot assays to assess binding specificity.

## 2 Materials and methods

### 2.1 CDR grafting

In this study, Complementarity-Determining Region (CDR) grafting was used to generate Nanobody (Wagner et al., 2018; Mirzaei et al., 2024; Reddy et al., 2024). We are using FDA-approved PD-1 inhibitors (Twomey and Zhang, 2021) for CDR grafting, specifically Pembrolizumab (IMGT/mAb DB ID – 472) (Kwok et al., 2016), Nivolumab (IMGT/mAb DB ID – 424) (Gunturi and McDermott, 2015), and Cemiplimab (IMGT/mAb DB ID – 846) (Markham and Duggan, 2018). Sequences for antibodies and Caplacizumab (IMGT/mAb DB ID – 401) Nanobody were obtained from the IMGT database (Manso et al., 2022). We selected Cemiplimab and Pembrolizumab for sequence alignment with Caplacizumab. The alignment indicated that Cemiplimab shared the highest similarity with Caplacizumab, reducing the likelihood of structural disruption during CDR grafting. Consequently, the CDRs from Cemiplimab were grafted onto the framework regions of Caplacizumab, whereas Nivolumab was excluded due to its association with cytokine release syndrome (Manso et al., 2022; Ciner et al., 2021). The newly designed nanobody was subjected to three-dimensional structural prediction using AlphaFold 2 (Mirdita et al., 2022), and its quality was assessed with a Ramachandran plot using the PROCHECK tool (Laskowski et al., 1993).

### 2.2 Physicochemical parameters analysis

The designed nanobody underwent thorough physicochemical analysis using multiple parameters. Initially, its molecular weight, theoretical isoelectric point (pI), stability index, aliphatic index, predicted half-life, and grand average of hydropathicity (GRAVY) were assessed with the ProtParam tool (Bidkar et al., 2014).

### 2.3 Antigenicity and allergy prediction

The tool Vaxigen v2.0 (Zaharieva et al., 2017) was employed to predict immunogenic epitopes within the Nanobody sequence, providing insights into potential immune responses upon administration. Furthermore, allergy prediction was conducted using the ALLERCATPRO 2 tool (Nguyen et al., 2022), which identifies allergenic regions within protein sequences.

### 2.4 PD-1/PD L-1 and PD-1/PD L-2 interaction analysis for epitope prdiction

The PD-1/PD-L1 and PD-1/PD L-2 interactions were analyzed by PBDsum online tool (Laskowski et al., 2018) utilizing the crystal structures of their complex obtained from the PDB with the ID 4ZQK, 6UMT (Zak et al., 2015; Wen et al., 2020; Tang and Kim, 2019). By examining this structural model, crucial insights into the binding interface and key amino acids involved in the PD-1/PD-L1 and PD-1/PD-L2 interactions were elucidated.

### 2.5 Molecular docking analysis

Molecular docking analysis was carried out using the ClusPro web tool (Desta et al., 2020; Vajda et al., 2017; Kozakov et al., 2013; Kozakov et al., 2017), where the PD-1 and PD-L1 complex (PDB ID: 4ZQK, Chain B) served as the receptor, and the designed nanobody was used as the ligand. A thorough exploration of ligand orientations was ensured by setting the number of ligand rotations to probe at 70,000. Post-docking, the structures interactions were analysed by using PDB sum webtool (Laskowski et al., 2018).

### 2.6 Molecular dynamic simulations of designed nanobody

We employed WEBGRO (Simlab, 2022) for macromolecular simulations to assess the stability of the constructed nanobody. The system was solvated with a water model, neutralized, and supplemented with 0.15 M NaCl, applying the GROMOS96 43a1 force field. Energy minimization was achieved in 5,000 steps using the steepest descent method. For equilibration, NVT and NPT simulations were performed. We conducted two 50 ns simulations, each with 1,000 frames, at temperatures of 300 K and 310 K (Poustforoosh et al., 2023). Both simulations were run at 1.0 bar. Stability was assessed through parameters including RMSD, RMSF, Rg, H-bonding, and SASA (Sultana et al., 2024).

### 2.7 Transformation and positive colony selection

The nanobody gene sequence, codon-optimized for *E. coli* expression, was synthesized and inserted into the pET-28a (+) expression vector (Synbio Technologies, NJ, United States) at the NdeI and XhoI restriction sites (Li et al., 2022). The recombinant vector was transformed into *Escherichia coli* BL21 (DE3) competent cells (Khirehgesh et al., 2021) using the heat shock method. Briefly, 50 µL of competent cells were mixed with 100 ng of the recombinant plasmid and incubated on ice for 30 min. The mixture was then subjected to heat shock at 42°C for 45 s, followed by rapid cooling on ice for 2 min. SOC medium (950 µL) was added to the cells, and the culture was incubated at 37°C for 1 h with shaking at 220 rpm (Chang et al., 2017).

Transformants were selected by plating on LB agar plates containing 50 μg/mL Kanamycin and incubated overnight at 37°C. Positive colonies were screened by colony PCR using primers specifically designed to amplify the inserted nanobody gene (Gussow and Clackson, 1989; Azevedo et al., 2017). Primers were designed using PrimerBLAST (NCBI) (Kozyreva et al., 2021), with sequences as follows:

Forward Primer: 5′CATTCGATGGTGTCCGGGAT-3'.

Reverse Primer: 5′-TCAGCTTCCTTTCGGGCTTT-3'.

PCR conditions were set as follows: initial denaturation at 95°C for 5 min, followed by 30 cycles of 95°C for 30 s, 53°C for 45 s, and 72°C for 1 min, with a final extension at 72°C for 5 min (Rychlik et al., 1990). PCR products were analyzed on a 1% agarose gel stained with ethidium bromide, and the expected amplicon size (nearly 825 bp) was confirmed by comparison with a DNA ladder and positive control (Amplification product of pure plasmid).

### 2.8 Production and purification of nanobody

A single positive colony was inoculated into 10 mL of LB broth containing 50 μg/mL Kanamycin and grown overnight at 37°C with shaking at 220 rpm. The overnight culture was then transferred into 1,000 mL of LB broth with Kanamycin and grown until the OD600 reached 0.6–0.8. Expression of the nanobody was induced by adding Isopropyl β-D-1-thiogalactopyranoside (IPTG) to a final concentration of 1 mM, followed by incubation at 18°C for 16 h with shaking at 180 rpm (Nikkhoi et al., 2017).

Cells were harvested by centrifugation at 6,000 × g for 10 min at 4°C, and the cell pellet was resuspended in lysis buffer (50 mM Tris -HCl, 150 mM NaCl, 1 mM PMSF, 10 mM imidazole, pH 8.0). The cells were lysed by sonication on ice, and the lysate was clarified by centrifugation at 12,000 × g for 30 min at 4°C. The nanobody was then purified using Ni-NTA agarose resin pre equilibrated using 50 mM Tris-HCl pH 8.0, 150 mM NaCl, 30 mM imidazole (Himedia, India). Bound proteins were eluted with elution buffer (50 mM Tris-HCl, 150 mM NaCl, 250 mM imidazole, pH 8.0), and fractions were analyzed by 15% SDS-PAGE. Eluted nanobody fractions were pooled and dialyzed overnight at 4°C in 50 mMTris-HCl, 150 mM NaCl, pH 8.0, with buffer changes to remove imidazole. After dialysis overnight the nanobody was subjected to protein estimation by the Lowry method to quantify protein concentration (Crowe et al., 1994; Zhang et al., 2020; Waterborg, 2009).

### 2.9 Western blotting and dot blotting for nanobody affinity towards PD-1

To assess the affinity of the nanobody for PD-1, both Western blotting and dot blot assays were conducted, with BSA included as a control in both assays. For Western blotting, human PD-1 Fc recombinant protein (Peprotech, United States, Catalog # 310–40-1 MG) and BSA were separated on a 12% SDS-PAGE gel and transferred onto a nitrocellulose membrane (Himedia, India). The membrane was blocked with 5% non-fat dry milk in TBST for 1 h at room temperature. After blocking, it was incubated overnight at 4°C with the purified nanobody as the primary antibody, diluted to 10 μg/mL in TBST (Zhang Y. et al., 2023; Shin et al., 2021).

For the dot blot, a series of PD-1 protein concentrations (200, 100, 50, 25 ng) and BSA as controls were directly spotted onto a nitrocellulose membrane, dried, and blocked similarly. The dot blot membrane was then incubated with the purified nanobody under the same conditions, allowing for a direct binding comparison across different PD-1 concentrations and the BSA control. Following primary incubation, both membranes (from Western and dot blot assays) were washed three times with TBST, then incubated with an anti-His tag HRP-conjugated secondary antibody (1:500 dilution, Invitrogen, United States, Cat no: MA1-21315-HRP) for 1 h at room temperature. After post-incubation washes, the blots were developed using tetramethylbenzidine (TMB) substrate solution (Himedia, India).

### 2.10 Enzyme-linked immunosorbent assay (ELISA) against PD-1

The binding activity of the purified nanobody against PD-1 was assessed by ELISA. Ninety-six-well plates were coated with 200 µL of 5 μg/mL recombinant human PD-1 in PBS and incubated overnight at 4°C. The plates were washed three times with PBS containing 0.05% Tween-20 (PBST) and blocked with 5% bovine serum albumin (BSA) in PBST for 1 h at room temperature. After blocking, 200 µL of serially diluted nanobody (starting from 1000 to 1.95 nM) was added to the wells and incubated for 2 h at room temperature. The plates were then washed with PBST and incubated with 200 µL of anti-His tag HRP-conjugated antibody (1:500 dilution, Invitrogen, United States, Cat no: MA1-21315-HRP) for 1 h. After washing, 200 µL of tetramethylbenzidine (TMB) substrate solution was added to each well, and the reaction was stopped with 100 µL of 2 M H2SO4. The absorbance was measured at 450 nm using a microplate reader (Benesphera E21) (Chen et al., 2020).

## 3 Results

### 3.1 CDR grafting and homology model building

In the CDR grafting process, the complementarity-determining regions (CDRs) from Cemiplimab were successfully grafted onto the framework regions of Caplacizumab. The resulting 3D model was constructed using AlphaFold2, a state-of-the-art protein structure prediction tool. The quality of the constructed nanobody model was rigorously evaluated using a Ramachandran plot, which is crucial for assessing the conformational angles of the protein backbone, ensuring that the model is structurally sound and free from steric clashes. The Ramachandran plot generated by PROCHECK demonstrated that 94.7% of the residues were located in the most favored regions, 5.3% in the additional allowed regions, with no residues in the generously allowed or disallowed regions. This indicates that the model is of high quality, with no significant structural issues. Specifically, the analysis included 95 non-glycine and non-proline residues, with 17 glycine residues and 3 proline residues, totaling 117 residues.

**FIGURE 1:**
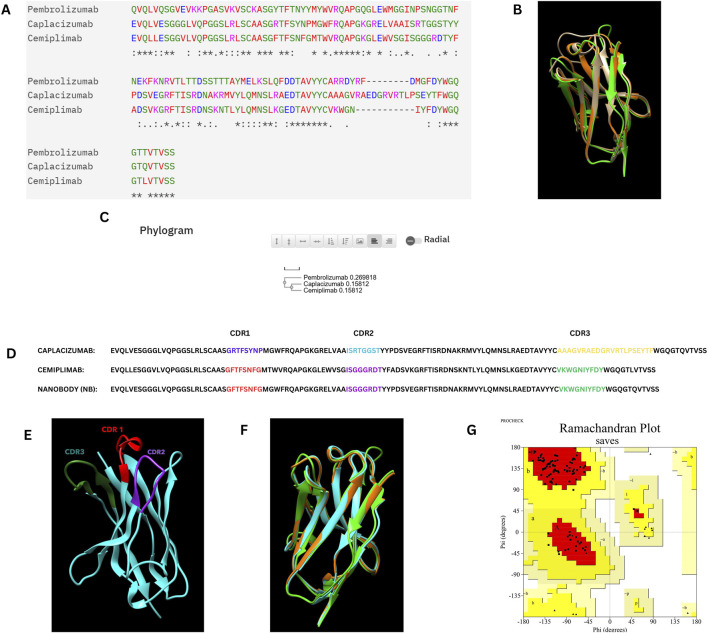
**(A)** Multiple sequence alignment of the Caplacizumab nanobody, Cemiplimab, and Pembrolizumab heavy chains. **(B)** Superimposition of the Caplacizumab nanobody (Brown), Cemiplimab (Oranage), and Pembrolizumab heavy chains (Green). **(C)** Phylogram of the Caplacizumab nanobody, Cemiplimab, and Pembrolizumab heavy chains. **(D)** Sequence alignment between Cemiplimab and Caplacizumab, with highlighted CDR grafting sequences in the designed nanobody. **(E)** Three-dimensional structure of the nanobody predicted by AlphaFold 2, with highlighted CDR sequences, visualized using UCSF Chimera. **(F)** Superimposition of the constructed nanobody (Cyan), Cemiplimab (Orange), and Pembrolizumab (Green) heavy chains. **(G)** Ramachandran plot of the nanobody structure, illustrating its conformational quality.

Figure 1 illustrates the overall process of multiple sequence alignment, CDR grafting and homology modeling, demonstrating the integration of Cemiplimab CDRs into the Caplacizumab framework. The resultant 3D structure generated by AlphaFold2 is visualized using UCSF Chimera (Pettersen et al., 2004), and the quality assessment is confirmed through the Ramachandran plot analysis.

### 3.2 Physicochemical analysis

The physicochemical attributes of the designed nanobody, analyzed by the ProtParam tool, revealed the following: a molecular weight (MW) of 12.5 kDa (with his tag ∼13.5 k Da), theoretical pI of 8.05, and instability index of 35.84, representing that the designed nanobody is stable. The aliphatic index was 64.96, and the estimated half-life in *E. coli* was greater than 10 h and the GRAVY was −0.257, which indicates nanobody potential suitability for practical applications. The number of negatively charged (Asp and Glu) amino acids was 10, while the number of positively charged (Arg and Lys) amino acids was 11.

### 3.3 Immunogenicity and allergy prediction

The immunogenicity and allergy potential of the nanobody were evaluated VaxiJen v2.0 and the ALLERCATPRO 2 tool, respectively. The average immunogenicity score was 0.59 (threshold value is 0.5), indicating that the nanobody is mild immunogenic and it has no allergy potential.

### 3.4 PD-1/PD-L1 and PD-1/PD L2 interaction analysis for epitope prediction

**FIGURE 2:**
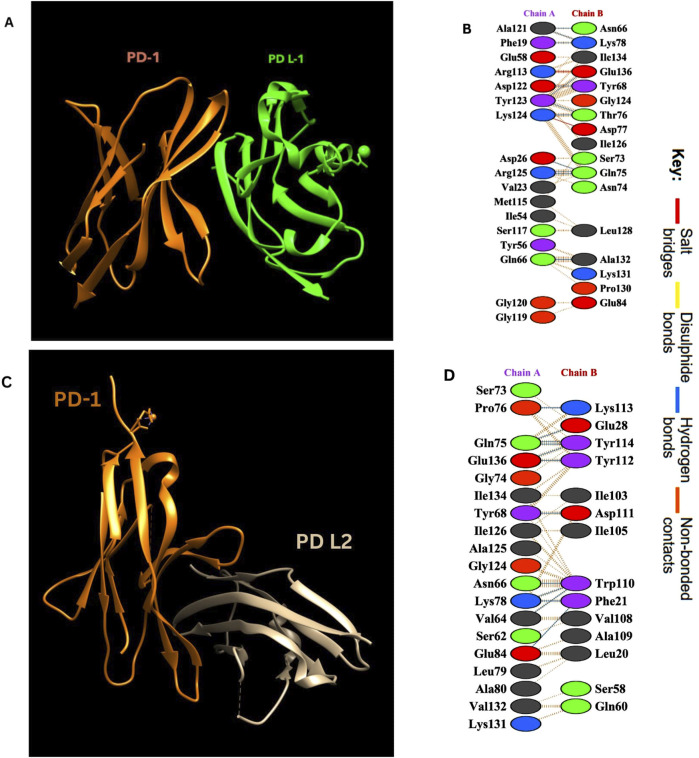
**(A)** PD-1/PD-L1 complex. Visualization of the complex was done by using UCSF Chimera (Pettersen et al., 2004). **(B)** Amino acid interactions between Chain A (PD-L1) and Chain B (PD-1). **(C)** PD-1/PD-L2 complex. **(D)** Amino acid interactions between Chain A (PD-1) and Chain B (PD- L2).

Interaction analysis of the PD-1/PD-L1 and PD-1/PD-L2 complexes using the PDBsum tool revealed detailed insights into the binding interface and key residues involved. The crystal structures (PDB ID: 4ZQK for PD-1/PD-L1 and 6UMT for PD-1/PD-L2) identified several hydrogen bonds, salt bridges, and non-bonded interactions stabilizing these complexes. Notably, residues spanning positions 65–85 and 124–136 in PD-1 were crucial in interacting with PD-L1. These interactions are summarized in Table 1 below and visualized in Figure 2, highlighting the molecular interactions of both complexes.

**TABLE 1:** Key interactions in PD-1/PD-l1 and PD-1/PD-l2 complexes.

Complex	Interaction type	Ligand	Receptor
PD-1/PD-L1 (PDB id: 4ZQK)	Salt bridges	Arg125, Asp126	Glu136, Asp77
	Hydrogen bonds	Ala121, Phe119, Gly58, Ser73, Met115, Ile54	Asn66, Tyr68, Ile134, Gln75, Ser73
	Non-bonded contacts	Tyr123, Val123, Gln66, Gly119	Leu128, Ala132, Pro130, Glu84
PD-1/PD-L2 (PDB id: 6UMT)	Salt bridges	Lys113	Glu134
	Hydrogen bonds	Tyr112, Glu28, Lys131	Pro76, Ser73, Glu136, Gly74, Gln75
	Non-bonded contacts	Asp111, Val108, Ala109, Leu20	Lys78, Glu84, Val64, Ala79, Glu87

### 3.5 Molecular docking study

**FIGURE 3:**
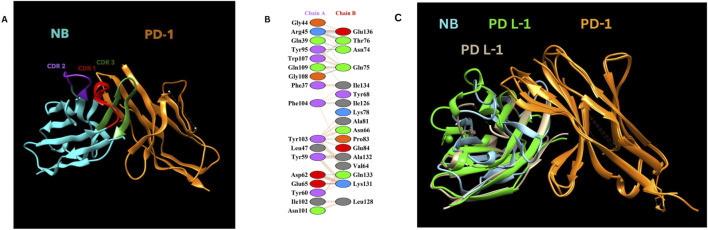
**(A)** NB and PD-1 docking structure. **(B)** Detailed amino acid interactions between Chain A (Constructed Nanobody as Ligand - NB) and Chain B (PD-1 as receptor). **(C)** Nanobody blocking PD-1/PD L-1 and PD-1/Pd L-2 interactions Visualization of the complex was done by using UCSF Chimera (Pettersen et al., 2004).

Molecular docking was performed using the ClusPro web tool, with the PD-1/PD-L1 complex (PDB ID: 4ZQK, Chain B, i. e., PD-1) as the receptor and the designed nanobody as the ligand. The best-docked complex demonstrated strong binding affinity, supported by multiple hydrogen bonds, salt bridges, and hydrophobic interactions at the binding interface. PDBsum analysis highlighted key residues involved in the interaction, summarized in Table 2 below. Figure 3 depicts the detailed molecular interactions between PD-1 and the nanobody, and elucidates the mechanism by which the nanobody inhibits the binding of PD-1 to its ligands, PD-L1 and PD-L2.

**TABLE 2:** Key interactions between the designed nanobody (chain A) and PD-1 (chain B).

Interaction type	Chain a (nanobody as ligand)	Chain B (PD-1 receptor)
Salt bridges	Asp62, Glu65	Lys131, Lys78
Hydrogen bonds	Gly44, Arg45, Gln39, Tyr95, Gln109, Tyr103, Leu47, Tyr59	Glu136, Thr76, Asn74, Gln75, Tyr68, Glu84, Ala132
Non-bonded contacts	Trp107, Gly108, Phe104, Ile102, Asn101	Gln133, Ile126, Ala81, Leu128

#### 3.6 Molecular dynamics simulation

**FIGURE 4:**
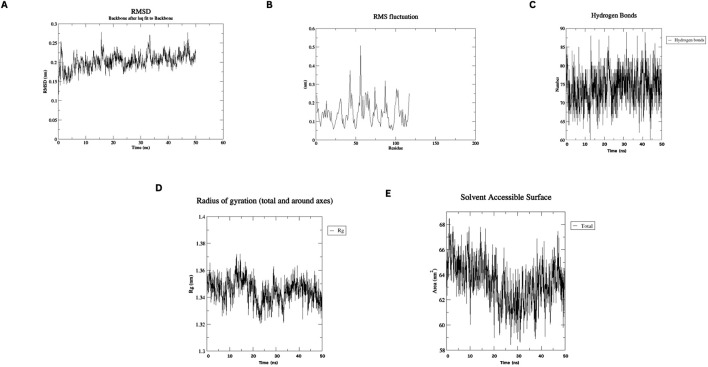
Molecular dynamics simulation plots at 300 K **(A)** RMSD analysis **(B)** RMSF analysis. **(C)** H-bonding analysis **(D)** Rg plot. **(E)** SASA assessment.

**FIGURE 5:**
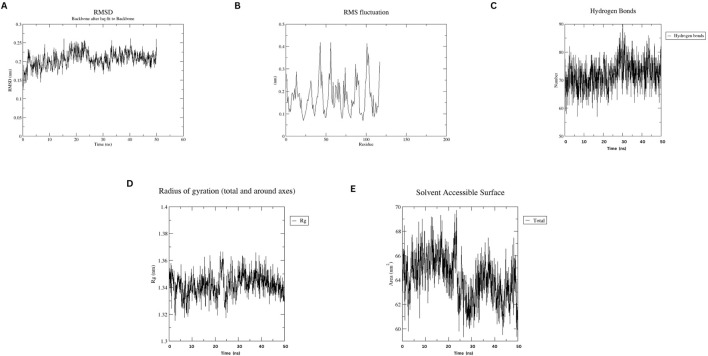
Molecular dynamics simulation plots at 310 K **(A)** RMSD analysis **(B)** RMSF analysis. **(C)** H-bonding analysis **(D)** Rg plot. **(E)** SASA assessment.

MD simulations of the designed nanobody revealed its stable structural behavior at 300 K and 310 K. RMSD analysis showed equilibration after ∼5 ns, stabilizing at ∼0.2 Å, indicating conformational stability even at elevated temperatures. RMSF analysis demonstrated minimal residue fluctuations, confirming structural integrity. Figures 4, 5 present the RMSD, RMSF, Radius of Gyration, H-bonds, and SASA data, highlighting the nanobody’s stability and dynamics under both conditions.

### 3.7 Transformation and colony PCR verification

The transformation of the pET-28a (+)-nanobody construct into *E. coli* BL21 (DE3) cells was successful, as indicated by the growth of kanamycin-resistant colonies. Positive colony screening was performed using colony PCR, with primers specific for the nanobody gene. The expected PCR product size was 825 bp, and agarose gel electrophoresis (figure provided in supplementary material) confirmed the presence of this product in selected colonies. This result confirms the successful insertion of the nanobody gene into the expression vector and its stable transformation into the bacterial host.

### 3.8 Expression and purification of the nanobody

**FIGURE 6:**
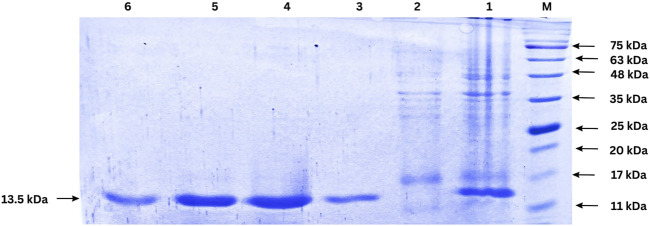
SDS-PAGE analysis of nanobody expression and purification. Lane M: Protein ladder; Lane 1: cell lysate after IPTG induction (1:50 dilution); Lane 2: Flow-through from Ni-NTA column (1:50 dilution); Lanes 3–6: Eluted fractions from Ni-NTA purification. The arrow indicates the purified nanobody band.

The expression of the nanobody was induced by IPTG in *E. coli* BL21 (DE3) cells, and the protein was successfully purified using Ni-NTA affinity chromatography. The purified protein was analyzed by SDS-PAGE, revealing a single band corresponding to the expected molecular weight of the nanobody, approximately 13.5 kDa (Figure 6). This result indicates successful expression, purification, and quantification of the nanobody. Protein quantification was performed after dialysis using the Lowry method, yielding approximately 1 mg of nanobody per liter of culture.

### 3.9 Western blotting for nanobody affinity towards PD-1

**FIGURE 7:**
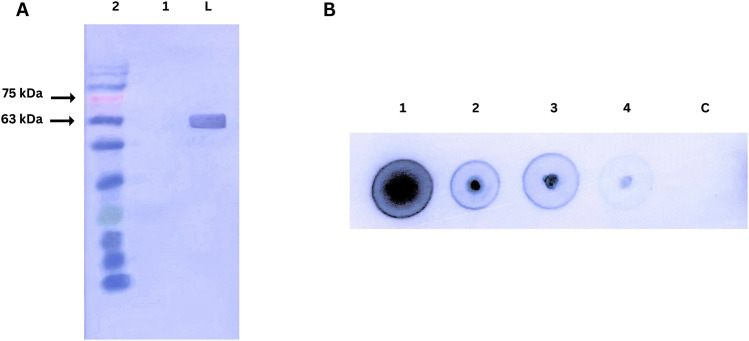
**(A)** Western blot analysis of nanobody affinity for PD-1. Lane M: Prestained protein ladder; Lane 1: Negative control (BSA); Lane 2: Human PD-1 protein probed with nanobody and detected with anti-His tag HRP-conjugated antibody. **(B)** Dot blot analysis of nanobody affinity for PD-1.1,2,3,4, C dots are 200ng, 100ng, 50ng, 25ng, Control (BSA 200 ng) respectively.

The affinity of the purified nanobody for PD-1 was assessed using Western blot and dot blot assays, as shown in Figure 7. In the Western blot, recombinant human PD-1 (200 ng) was separated by SDS-PAGE, transferred to a nitrocellulose membrane, and probed with the nanobody. A strong signal at ∼63 kDa, detected using an anti-His tag HRP-conjugated antibody, confirmed the nanobody’s specific binding to denatured PD-1.

The dot blot assay evaluated concentration-dependent binding by spotting varying amounts of PD-1 on a nitrocellulose membrane. Strong signals across the protein gradient demonstrated the nanobody’s effective binding to native PD-1. A BSA control confirmed specificity, highlighting the nanobody’s integrity and functionality in both assays.

### 3.10 Binding activity of the nanobody to PD-1 by ELISA

**FIGURE 8:**
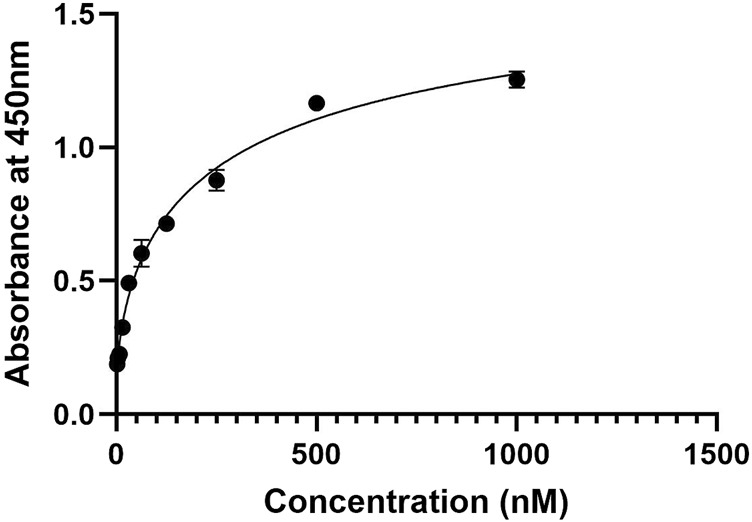
ELISA binding curve of the nanobody against PD-1, showing absorbance at 450 nm across nanobody concentrations from 1.95 nM to 1,000 nM. The curve demonstrates a saturation pattern, indicating high-affinity binding to PD-1.

The binding affinity of the developed nanobody against PD-1, ELISA assay was performed (Figure 8) with a series of nanobody concentrations ranging from 1,000 to 1.95 nM The absorbance at 450 nm increased with nanobody concentration, indicating specific binding to PD-1. A saturation curve was observed, suggesting a high-affinity interaction between the nanobody and PD-1. At lower concentrations (1.95–125 nM), there was a gradual increase in absorbance, while higher concentrations (500 nM and above) approached a plateau, indicating near-maximal binding. These results demonstrate that the nanobody binds to PD-1 with high affinity, validating its potential use for potential therapeutic applications.

## 4 Discussion

Immunotherapy has revolutionized cancer treatment by leveraging the body’s immune system to target and eliminate tumor cells, offering advantages such as reduced systemic toxicity and the ability to achieve long-term remission compared to traditional therapies like chemotherapy (Sharma and Allison, 2015). Monoclonal antibodies (mAbs) have been central to this success, particularly in targeting immune checkpoints like PD-1. However, they often come with limitations including high production costs, risk of immunogenicity, and complex tissue penetration profiles (Michot et al., 2016). In this context, nanobodies emerge as crucial alternatives due to their smaller size, ease of production, and reduced immunogenicity (Ewert et al., 2002). For example, Caplacizumab, a nanobody against von Willebrand factor, has demonstrated rapid and effective outcomes in treating thrombotic thrombocytopenic purpura with fewer adverse reactions, underscoring the potential of nanobodies in immunotherapy (Scully et al., 2019).

Our designed nanobody targeting PD-1, developed by grafting Cemiplimab CDRs onto Caplacizumab FR regions, presents favorable characteristics for immunotherapeutic use. With a molecular weight of 12.5 kDa, it is significantly smaller than conventional mAbs like Cemiplimab and Pembrolizumab, which are around 150 kDa. This size reduction could enhance tissue penetration and clearance rates. The theoretical pI of 8.05 and a stability-indicating instability index of 35.84 suggest that our nanobody is well-suited for physiological conditions without requiring extensive stabilization, unlike some mAbs. The nanobody’s moderate hydrophobicity, reflected by an aliphatic index of 64.96 and a GRAVY score of −0.257, supports its solubility and structural integrity. Its estimated half-life exceeding 10 h in *E. coli* hints at efficient and scalable production capabilities. Additionally, the average immunogenicity score of 0.471 indicates low potential for immune responses, and its non-allergenic profile compares favorably against mAbs, which often need humanization to reduce immunogenicity. Molecular dynamics simulations at 300 and 310 K show that the nanobody maintains stable conformation with minimal RMSD and RMSF fluctuations, highlighting its robustness under physiological and slightly elevated temperatures. This structural stability contrasts with some antibodies that exhibit conformational variability. Docking analysis identified various interaction types between the Nanobody (Chain A) and the PD-1 receptor (Chain B). Salt bridges were observed between Asp62 and Glu65 of the Nanobody and Lys131 and Lys78 of PD-1. Hydrogen bonds involved multiple residues, including Gly44, Arg45, Gln39, Tyr95, Gln109, Tyr103, Leu47, and Tyr59 on the Nanobody, and Glu136, Thr76, Asn74, Gln75, Tyr68, Glu84, and Ala132 on PD-1. Additionally, non-bonded contacts were formed by Trp107, Gly108, Phe104, Ile102, and Asn101 from the Nanobody, interacting with Gln133, Ile126, Ala81, and Leu128 of PD-1. These interactions collectively stabilize the complex and disrupt the PD-1/PD-L1 and PD-1/PD-L2 interactions.

Experimental characterization of the nanobody further reinforced these computational findings. Following production and purification, Western blot and ELISA assays demonstrated nanobody binding affinity for PD-1, supporting its functional viability. Dot blot assays assessed binding specificity, highlighting the nanobody precision in targeting PD-1. The nanobody’s efficacy in disrupting PD-1/PD-L1 binding, combined with its structural stability, low immunogenicity, and production advantages, suggests a promising therapeutic profile that could offer benefits over traditional antibody-based therapies. Interestingly, Poustforoosh et al. (2023) developed a nanobody targeting CD20 and assessed comparable parameters, finding values near to ours (Poustforoosh et al., 2023). This parallel research reinforces the robustness and potential of our designed nanobody. Mohammad Mehdi Heidari et al. developed a nanobody targeting CD20. However, in our nanobody, the tyrosine’s in the CDR3 sequence form hydrogen bonds with PD-1 amino acids (Heidari et al., 2024). While these tools provide critical insights into nanobody design, we note that they may not fully account for the complexities of biological systems. Experimental validation in advanced animal models or clinical settings would be a necessary step for future studies.

In summary, our nanobody not only effectively targets PD-1 but also brings significant advantages in stability, production, and safety, positioning it as a potent and innovative alternative in the landscape of immunotherapy.

## 5 Conclusion

This study presents a successful design, experimental validation, and evaluation of a nanobody targeting PD-1, combining advanced *in silico* techniques with laboratory characterization. Through CDR grafting, AlphaFold2-based homology modeling, MD simulations, and molecular docking, the nanobody was shown to have favorable physicochemical properties, low immunogenicity, and strong potential to block the PD-1/PD-L1 and PD-1/PD-L2 interactions, a critical pathway in cancer immunotherapy. Key residues, including Tyr59, Tyr60, Asp62, and Glu65 from the nanobody and Lys131, Gln133, and Leu128 from PD-1, were identified as critical for forming stable interactions, further enhancing its inhibitory efficacy. MD simulations confirmed the nanobody’s stability, supporting its potential as a therapeutic candidate. Experimental validation reinforced these computational findings, with Western blotting and ELISA demonstrating high binding affinity for PD-1, while dot blot assays confirmed its binding specificity. These combined results highlight the designed nanobody’s ability to enhance anti-tumor immunity and provide a strong foundation for future clinical development in cancer therapy.

## Data Availability

The original contributions presented in the study are included in the article/supplementary material, further inquiries can be directed to the corresponding authors.

## Abbreviations

CDR, Complementarity-Determining Region; FDA, Food and Drug Administration, United States; GRAVY, Grand Average of Hydropathicity; H-bonds, Hydrogen Bonding; MD, Molecular Dynamics; MW, Molecular Weight; NB, Nanobody; NVT, Constant volume and Temperature; NPT, Constant pressure and Temperature; pI, Isoelectric Point; PCR, Polymerase chain reaction; PD-1, Programmed Death Protein-1; PDB, Protein Data Bank; PD-L1, Programmed Death Ligand-1; Rg, Radius of Gyration; RMSD, Root Mean Square Deviation; RMSF, Root Mean Square Fluctuation; SASA, Solvent Accessible Surface Area.

## References

[B1] Alturki N. A. (2023). Review of the immune checkpoint inhibitors in the context of cancer treatment. J. Clin. Med. 12 (13), 4301. 10.3390/jcm12134301 37445336 PMC10342855

[B2] Azevedo F. Pereira H. Johansson B. (2017). Colony PCR. PCR Methods Protoc. 1620, 129–139. 10.1007/978-1-4939-7060-5_8 28540704

[B3] Bannas P. Hambach J. Koch-Nolte F. (2017). Nanobodies and nanobody-based human heavy chain antibodies as antitumor therapeutics. Front. Immunol. 8, 1603. 10.3389/fimmu.2017.01603 29213270 PMC5702627

[B4] Bidkar A. Thakur N. Bolshette J. D. Gogoi R. (2014). In-silico structural and functional analysis of hypothetical proteins of leptospira interrogans. Biochem. Pharmacol. 3 (136), 2167–0501. 10.4172/2167-0501.1000136

[B5] Chang A. Y. Chau V. Landas J. A. Pang Y. (2017). Preparation of calcium competent *Escherichia coli* and heat-shock transformation. JEMI methods 1 (22-25).

[B6] Chen J. Zhao Y. Feng W. (2020). Selection, preparation and characterization of scFv against human lipocalin 6 by phage display technology. Protein Expr. Purif. 171, 105627. 10.1016/j.pep.2020.105627 32205279

[B7] Ciner A. T. Hochster H. S. August D. A. Carpizo D. R. Spencer K. R. (2021). Delayed cytokine release syndrome after neoadjuvant nivolumab: a case report and literature review. Immunotherapy 13 (13), 1071–1078. 10.2217/imt-2020-0329 34287029 PMC8656293

[B8] Crowe J. Dobeli H. Gentz R. Hochuli E. Stiiber D. Henco K. (1994). 6xHis-Ni-NTA chromatography as a superior technique in recombinant protein expression/purification. Protoc. gene analysis 31, 371–387. 10.1385/0-89603-258-2:371 7921034

[B9] de Miguel M. Calvo E. (2020). Clinical challenges of immune checkpoint inhibitors. Cancer Cell 38 (3), 326–333. 10.1016/j.ccell.2020.07.004 32750319

[B10] Desta I. T. Porter K. A. Xia B. Kozakov D. Vajda S. (2020). Performance and its limits in rigid body protein-protein docking. Structure. 28 (9), 1071–1081.e3. 10.1016/j.str.2020.06.006 32649857 PMC7484347

[B11] Ewert S. Cambillau C. Conrath K. Plückthun A. (2002). Biophysical properties of camelid VHH domains compared to those of human VH3 domains. Biochemistry 41 (11), 3628–3636. 10.1021/bi011239a 11888279

[B12] Farkona S. Eleftherios P. D. Blasutig I. M. (2016). Cancer immunotherapy: the beginning of the end of cancer? BMC Med. 14, 1–18. 10.1186/s12916-016-0623-5 27151159 PMC4858828

[B13] Fife B. T. Pauken K. E. (2011). The role of the PD‐1 pathway in autoimmunity and peripheral tolerance. Ann. N. Y. Acad. Sci. 1217 (1), 45–59. 10.1111/j.1749-6632.2010.05919.x 21276005

[B14] Gasteiger E. Hoogland C. Gattiker A. Duvaud S. E. Wilkins M. R. Appel R. D. (2005). Protein identification and analysis tools on the ExPASy server. Geneva, Switzerland: Swiss Institute of Bioinformatics, 571–607.

[B15] Gunturi A. McDermott D. F. (2015). Nivolumab for the treatment of cancer. Expert Opin. investigational drugs 24 (2), 253–260. 10.1517/13543784.2015.991819 25494679

[B16] Gussow D. Clackson T. (1989). Direct clone characterization from plaques and colonies by the polymerase chain reaction. Nucleic Acids Res. 17 (10), 4000. 10.1093/nar/17.10.4000 2734114 PMC317898

[B17] Hashemi Z. S. Zarei M. Fath M. K. Ganji M. Farahani M. S. Afsharnouri F. (2021). *In silico* approaches for the design and optimization of interfering peptides against protein–protein interactions. Front. Mol. Biosci. 8, 669431. 10.3389/fmolb.2021.669431 33996914 PMC8113820

[B18] Heidari M. M. Shirazi E. A. Cheraghi S. F. Shahshahani R. Rahnama T. Khatami M. (2024). CDR grafting and site-directed mutagenesis approach for the generation and affinity maturation of Anti-CD20 nanobody. Mol. Biol. Rep. 51 (1), 751. 10.1007/s11033-024-09684-2 38874667

[B19] Jin B.-K. Odongo S. Radwanska M. Magez S. (2023). NANOBODIES®: a review of diagnostic and therapeutic applications. Int. J. Mol. Sci. 24 (6), 5994. 10.3390/ijms24065994 36983063 PMC10057852

[B20] Khirehgesh M. R. Sharifi J. Akbari B. Mansouri K. Safari F. Soleymani B. (2021). Design and construction a novel humanized biparatopic nanobody-based immunotoxin against epidermal growth factor receptor (EGFR). J. Drug Deliv. Sci. Technol. 66, 102837. 10.1016/j.jddst.2021.102837

[B21] Kozakov D. Beglov D. Bohnuud T. Mottarella S. Xia B. Hall D. R. (2013). How good is automated protein docking? Proteins Struct. Funct. Bioinforma. 81 (12), 2159–2166. 10.1002/prot.24403 PMC3934018 23996272

[B22] Kozakov D. Hall D. R. Xia B. Porter K. A. Padhorny D. Yueh C. (2017). The ClusPro web server for protein-protein docking. Nat. Protoc. 12 (2), 255–278. 10.1038/nprot.2016.169 28079879 PMC5540229

[B23] Kozyreva A. A. Zlotina A. M. Golovkin A. S. Kalinina O. V. Kostareva A. A. (2021). Primer designing in primer-BLAST. Transl. Med. 8 (3), 37–52. 10.18705/2311-4495-2021-8-3-37-52

[B24] Kuchroo J. R. Hafler D. A. Sharpe A. H. Lucca L. E. (2021). The double-edged sword: harnessing PD-1 blockade in tumor and autoimmunity. Sci. Immunol. 6 (65), eabf4034. 10.1126/sciimmunol.abf4034 34739340 PMC13541067

[B25] Kwok G. Yau T. C. C. Chiu J. W. Tse E. Kwong Y. L. (2016). Pembrolizumab (keytruda). Hum. vaccines and Immunother. 12 (11), 2777–2789. 10.1080/21645515.2016.1199310 PMC5137544 27398650

[B26] Laskowski R. A. Jabłońska J. Pravda L. Vařeková R. S. Thornton J. M. (2018). PDBsum: structural summaries of PDB entries. Protein Sci. 27 (1), 129–134. 10.1002/pro.3289 28875543 PMC5734310

[B27] Laskowski R. A. MacArthur M. W. Moss D. S. Thornton J. M. (1993). PROCHECK - a program to check the stereochemical quality of protein structures. J. App. Cryst. 26, 283–291. 10.1107/s0021889892009944

[B28] Li C. Zhang N. Zhou J. Ding C. Jin Y. Cui X. (2018). Peptide blocking of PD-1/PD-L1 interaction for cancer immunotherapy. Cancer Immunol. Res. 6 (2), 178–188. 10.1158/2326-6066.cir-17-0035 29217732

[B29] Li L. Li H. Tian Q. Ge B. Xu X. Chi Y. (2022). Expression and purification of soluble recombinant β-lactamases using *Escherichia coli* as expression host and pET-28a as cloning vector. Microb. Cell Factories 21 (1), 244. 10.1186/s12934-022-01972-5 PMC9686023 36419169

[B30] Liu J. Chen Z. Li Y. Zhao W. Wu J. Zhang Z. (2021a). PD-1/PD-L1 checkpoint inhibitors in tumor immunotherapy. Front. Pharmacol. 12, 731798. 10.3389/fphar.2021.731798 34539412 PMC8440961

[B31] Liu M. Li L. Jin D. Liu Y. (2021b). Nanobody—a versatile tool for cancer diagnosis and therapeutics. Wiley Interdiscip. Rev. Nanomedicine Nanobiotechnology 13 (4), e1697. 10.1002/wnan.1697 33470555

[B32] Manso T. Folch G. Giudicelli V. Jabado-Michaloud J. Kushwaha A. Nguefack Ngoune V. (2022). IMGT® databases, related tools and web resources through three main axes of research and development. Nucleic acids Res. 50 (D1), D1262–D1272. 10.1093/nar/gkab1136 34875068 PMC8728119

[B33] Markham A. Duggan S. (2018). Cemiplimab: first global approval. Drugs 78 (17), 1841–1846. 10.1007/s40265-018-1012-5 30456447

[B34] Michot J. M. Bigenwald C. Champiat S. Collins M. Carbonnel F. Postel-Vinay S. (2016). Immune-related adverse events with immune checkpoint blockade: a comprehensive review. Eur. J. cancer 54, 139–148. 10.1016/j.ejca.2015.11.016 26765102

[B35] Mirdita M. Schütze K. Moriwaki Y. Heo L. Ovchinnikov S. Steinegger M. (2022). ColabFold: making protein folding accessible to all. Nat. methods 19 (6), 679–682. 10.1038/s41592-022-01488-1 35637307 PMC9184281

[B36] Mirzaei M. Mirhoseini S. Heidari M. M. Khatami M. (2024). Design and production of a novel anti-PD-1 nanobody by CDR grafting and site-directed mutagenesis approach. Mol. Biotechnol., 1–9. 10.1007/s12033-024-01162-1 38736021

[B37] Nguyen M. N. Krutz N. L. Limviphuvadh V. Lopata A. L. Gerberick G. Maurer-Stroh S. (2022). AllerCatPro 2.0: a web server for predicting protein allergenicity potential. Nucleic Acids Res. 50 (W1), W36–W43. 10.1093/nar/gkac446 35640594 PMC9252832

[B38] Nikkhoi S. K. Rahbarizadeh F. Ahmadvand D. (2017). Oligo-clonal nanobodies as an innovative targeting agent for cancer therapy: new biology and novel targeting systems. Protein Expr. Purif. 129, 115–121. 10.1016/j.pep.2016.09.012 27693491

[B39] Pettersen E. F. Goddard T. D. Huang C. C. Couch G. S. Greenblatt D. M. Meng E. C. (2004). UCSF Chimera—a visualization system for exploratory research and analysis. J. Comput. Chem. 25 (13), 1605–1612. 10.1002/jcc.20084 15264254

[B40] Poustforoosh A. Faramarz S. Negahdaripour M. Hashemipour H. (2023). Modeling and affinity maturation of an anti-CD20 nanobody: a comprehensive in-silico investigation. Sci. Rep. 13 (1), 582. 10.1038/s41598-023-27926-4 36631511 PMC9834265

[B41] Reddy D. J. Guntuku G. Palla M. S. (2024). Advancements in nanobody generation: integrating conventional, *in silico*, and machine learning approaches. Biotechnol. Bioeng. 121, 3375–3388. 10.1002/bit.28816 39054738

[B42] Rychlik W. J. S. W. Spencer W. J. Rhoads R. E. (1990). Optimization of the annealing temperature for DNA amplification *in vitro* . Nucleic acids Res. 18 (21), 6409–6412. 10.1093/nar/18.21.6409 2243783 PMC332522

[B43] Scully M. Cataland S. R. Peyvandi F. Coppo P. Knöbl P. Kremer Hovinga J. A. (2019). Caplacizumab treatment for acquired thrombotic thrombocytopenic purpura. N. Engl. J. Med. 380 (4), 335–346. 10.1056/nejmoa1806311 30625070

[B44] Sharma P. Allison J. P. (2015). Immune checkpoint targeting in cancer therapy: toward combination strategies with curative potential. Cell 161 (2), 205–214. 10.1016/j.cell.2015.03.030 25860605 PMC5905674

[B45] Shin J. Phelan P. J. Gjoerup O. Bachovchin W. Bullock P. A. (2021). Characterization of a single chain variable fragment of nivolumab that targets PD-1 and blocks PD-L1 binding. Protein Expr. Purif. 177, 105766. 10.1016/j.pep.2020.105766 32987122 PMC7518118

[B46] Shiravand Y. Khodadadi F. Kashani S. M. A. Hosseini-Fard S. R. Hosseini S. Sadeghirad H. (2022). Immune checkpoint inhibitors in cancer therapy. Curr. Oncol. 29 (5), 3044–3060. 10.3390/curroncol29050247 35621637 PMC9139602

[B47] Simlab (2022). WebGRO for macromolecular simulations. Little Rock, AR: The University of Arkansas for Medical Sciences (UAMS). Available at: https://simlab.uams.edu/(Accessed July 8, 2024).

[B48] Stirling E. R. Bronson S. M. Mackert J. D. Cook K. L. Triozzi P. L. Soto-Pantoja D. R. (2022). Metabolic implications of immune checkpoint proteins in cancer. Cells 11 (1), 179. 10.3390/cells11010179 35011741 PMC8750774

[B49] Sultana T. Mou S. I. Chatterjee D. Faruk M. O. Hosen M. I. (2024). Computational exploration of SLC14A1 genetic variants through structure modeling, protein-ligand docking, and molecular dynamics simulation. Biochem. Biophysics Rep. 38, 101703. 10.1016/j.bbrep.2024.101703 PMC11001776 38596408

[B50] Tang S. Kim P. S. (2019). A high-affinity human PD-1/PD-L2 complex informs avenues for small-molecule immune checkpoint drug discovery. Proc. Natl. Acad. Sci. 116 (49), 24500–24506. 10.1073/pnas.1916916116 31727844 PMC6900541

[B51] Twomey J. D. Zhang B. (2021). Cancer immunotherapy update: FDA-approved checkpoint inhibitors and companion diagnostics. AAPS J. 23, 39–11. 10.1208/s12248-021-00574-0 33677681 PMC7937597

[B52] Vajda S. Yueh C. Beglov D. Bohnuud T. Mottarella S. E. Xia B. (2017). New additions to the ClusPro server motivated by CAPRI. Proteins Struct. Funct. Bioinforma. 85 (3), 435–444. 10.1002/prot.25219 PMC5313348 27936493

[B53] Van Audenhove I. Gettemans J. (2016). Nanobodies as versatile tools to understand, diagnose, visualize and treat cancer. EBioMedicine 8, 40–48. 10.1016/j.ebiom.2016.04.028 27428417 PMC4919472

[B54] Wagner H. J. Wehrle S. Weiss E. Cavallari M. Weber W. (2018). A two-step approach for the design and generation of nanobodies. Int. J. Mol. Sci. 19 (11), 3444. 10.3390/ijms19113444 30400198 PMC6274671

[B55] Waterborg J. H. (2009). The Lowry method for protein quantitation. protein Protoc. Handb., 7–10. 10.1007/978-1-59745-198-7_2

[B56] Wen B. Zhao L. Wang Y. Qiu C. Xu Z. Huang K. (2020). Nanobodies targeting the interaction interface of programmed death receptor 1 (PD-1)/PD-1 ligand 1 (PD-1/PD-L1). Prep. Biochem. and Biotechnol. 50 (3), 252–259. 10.1080/10826068.2019.1692217 31799894

[B57] Yi M. Zheng X. Niu M. Zhu S. Ge H. Wu K. (2022). Combination strategies with PD-1/PD-L1 blockade: current advances and future directions. Mol. cancer 21.1, 28. 10.1186/s12943-021-01489-2 35062949 PMC8780712

[B58] Yu S. Xiong G. Zhao S. Tang Y. Tang H. Wang K. (2021). Nanobodies targeting immune checkpoint molecules for tumor immunotherapy and immunoimaging (Review). Int. J. Mol. Med. 47 (2), 444–454. 10.3892/ijmm.2020.4817 33416134 PMC7797440

[B59] Zaharieva N. Dimitrov I. Flower D. Doytchinova I. (2017). Immunogenicity prediction by VaxiJen: a ten year overview. J. Proteom. Bioinform 10 (11), 10–4172. 10.4172/jpb.1000454

[B60] Zak K. M. Kitel R. Przetocka S. Golik P. Guzik K. Musielak B. (2015). Structure of the complex of human programmed death 1, PD-1, and its ligand PD-L1. Structure 23 (12), 2341–2348. 10.1016/j.str.2015.09.010 26602187 PMC4752817

[B61] Zhang Y. Yang S. Jiang D. Li Y. Ma S. Wang L. (2023a). Screening and identification of an anti-PD-1 nanobody with antitumor activity. Biosci. Rep. 43 (1), BSR20221546. 10.1042/bsr20221546 PMC9867944 36475449

[B62] Zhang Y. Yang S. Jiang D. Li Y. Ma S. Wang L. (2023b). Screening and identification of an anti-PD-1 nanobody with antitumor activity. Biosci. Rep. 43 (1), BSR20221546. 10.1042/bsr20221546 PMC9867944 36475449

[B63] Zhang Z. Wang Y. Ding Y. Hattori M. (2020). Structure-based engineering of anti-GFP nanobody tandems as ultra-high-affinity reagents for purification. Sci. Rep. 10 (1), 6239. 10.1038/s41598-020-62606-7 32277083 PMC7148334

