# Supplementary material for: Innovative CDR grafting and computational methods for PD-1 specific nanobody design
Source: Front Bioinform. 2025 Jan 17;4:1488331. doi: 10.3389/fbinf.2024.1488331 (PMC11782559; doi:10.3389/fbinf.2024.1488331)

Webtools used for this research paper

| S.No | Web tool | URL |
| --- | --- | --- |
| 1. | IMGT | <https://www.imgt.org/> |
| 2. | AlphaFold 2 | <https://colab.research.google.com/github/sokrypton/ColabFold/blob/main/AlphaFold2.ipynb> |
| 3. | PROCHECK | <https://saves.mbi.ucla.edu/> |
| 4. | ProtParam | <https://web.expasy.org/protparam/> |
| 5. | VaxiJen 2.0 server | <https://www.ddg-pharmfac.net/vaxijen/VaxiJen/VaxiJen.html> |
| 6. | ALLERCATPRO 2 | <https://allercatpro.bii.a-star.edu.sg/> |
| 7. | PBDsum | <https://www.ebi.ac.uk/thornton-srv/databases/pdbsum/Generate.html> |
| 8. | WEBGRO | <https://simlab.uams.edu/> |
| 9. | Cluspro | <https://cluspro.bu.edu/login.php> |
| 10. | UCSF Chimera | <https://www.rbvi.ucsf.edu/chimera/> |
|  |  |  |

Colony PCR

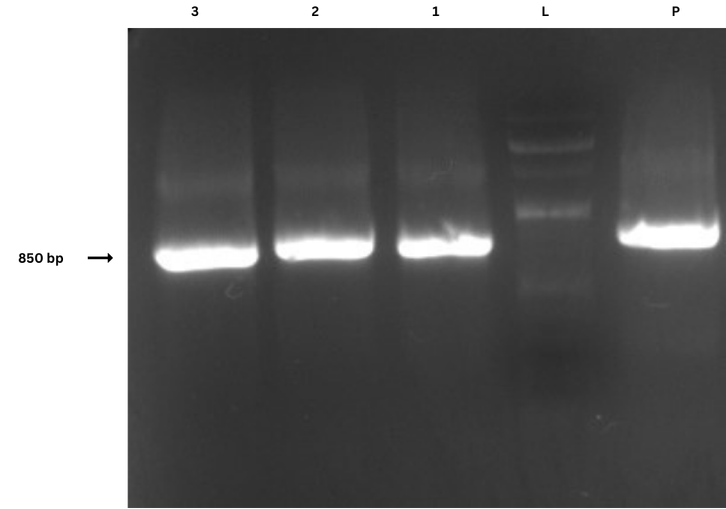


825 bp

Colony PCR for selected colonies. Lane L: 1 Kb Ladder, Lane P: Plasmid, Lane 1,2,3 are individual colonies on kanamycin plate.

Insilico pcr results: (Performed on JAVA in house script)
Input gene sequence and primers:


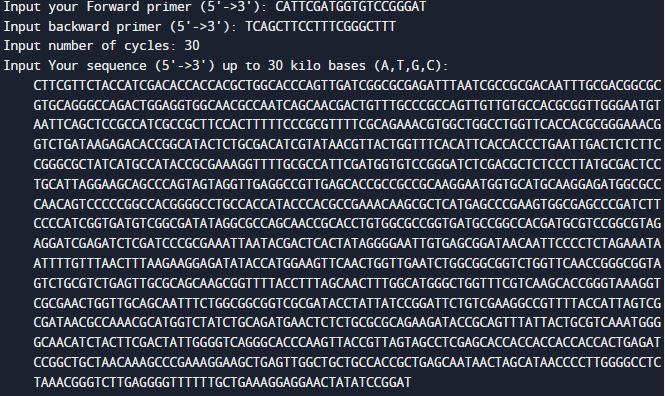


Output:


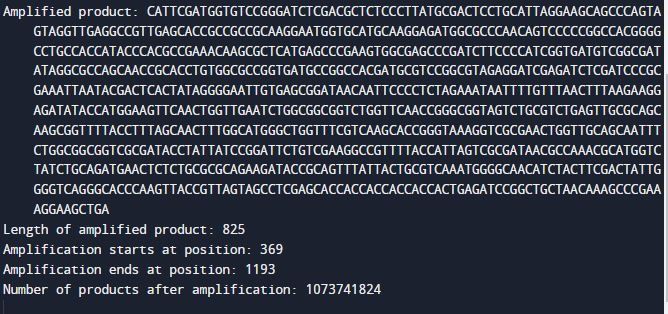


ELISA results:

| **Concentarion (nM)** | **Absorbence at 450** | |
| --- | --- | --- |
| 1000 | 1.226 | 1.233 |
| 500 | 1.112 | 1.138 |
| 250 | 0.881 | 0.847 |
| 125 | 0.751 | 0.648 |
| 62.5 | 0.713 | 0.634 |
| 31.25 | 0.465 | 0.479 |
| 15.63 | 0.27 | 0.357 |
| 7.81 | 0.23 | 0.258 |
| 3.9 | 0.198 | 0.198 |
| 1.95 | 0.191 | 0.186 |
| Control | 0.070 | 0.080 |

Interaction map of PD – 1 (Blue) / PD L-1 (Pink) using Icn3D:


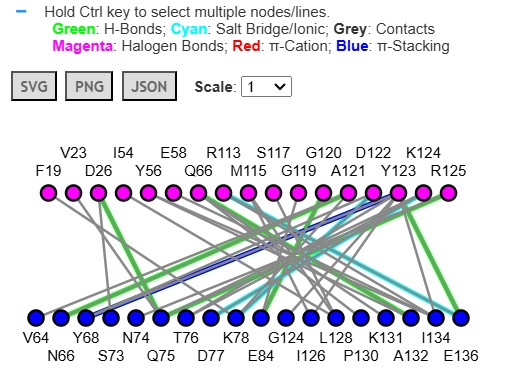

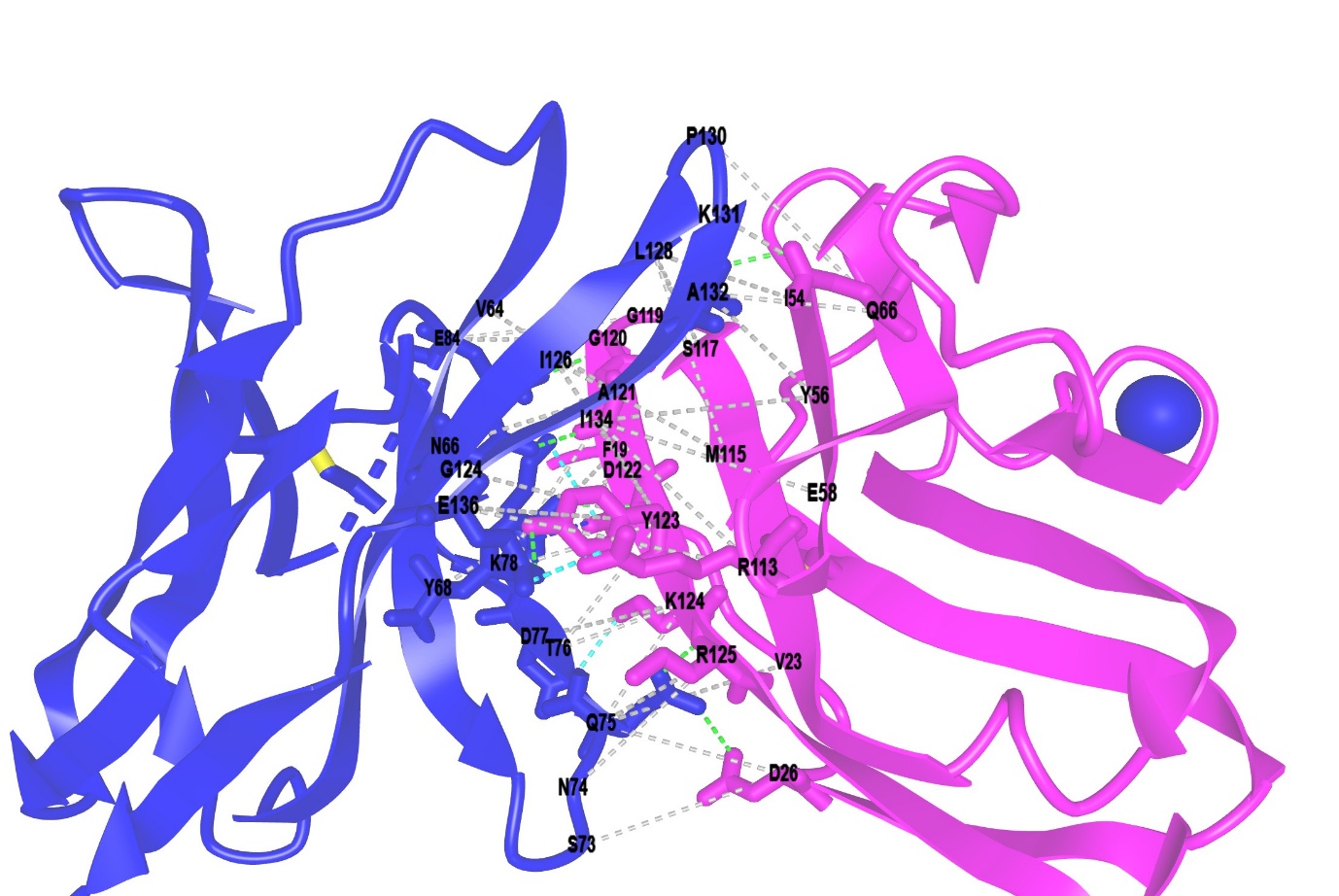


Interaction map of PD -1 (Pink) / PD L-2 (Blue) using Icn3D:


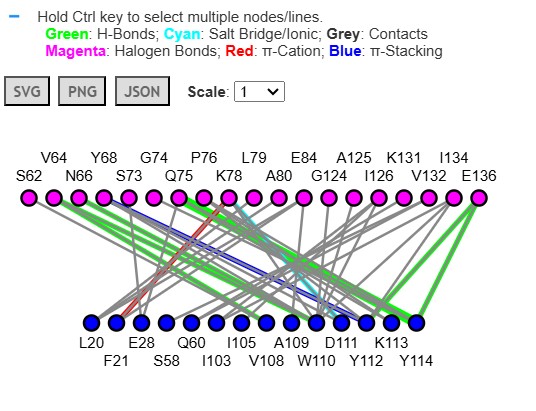

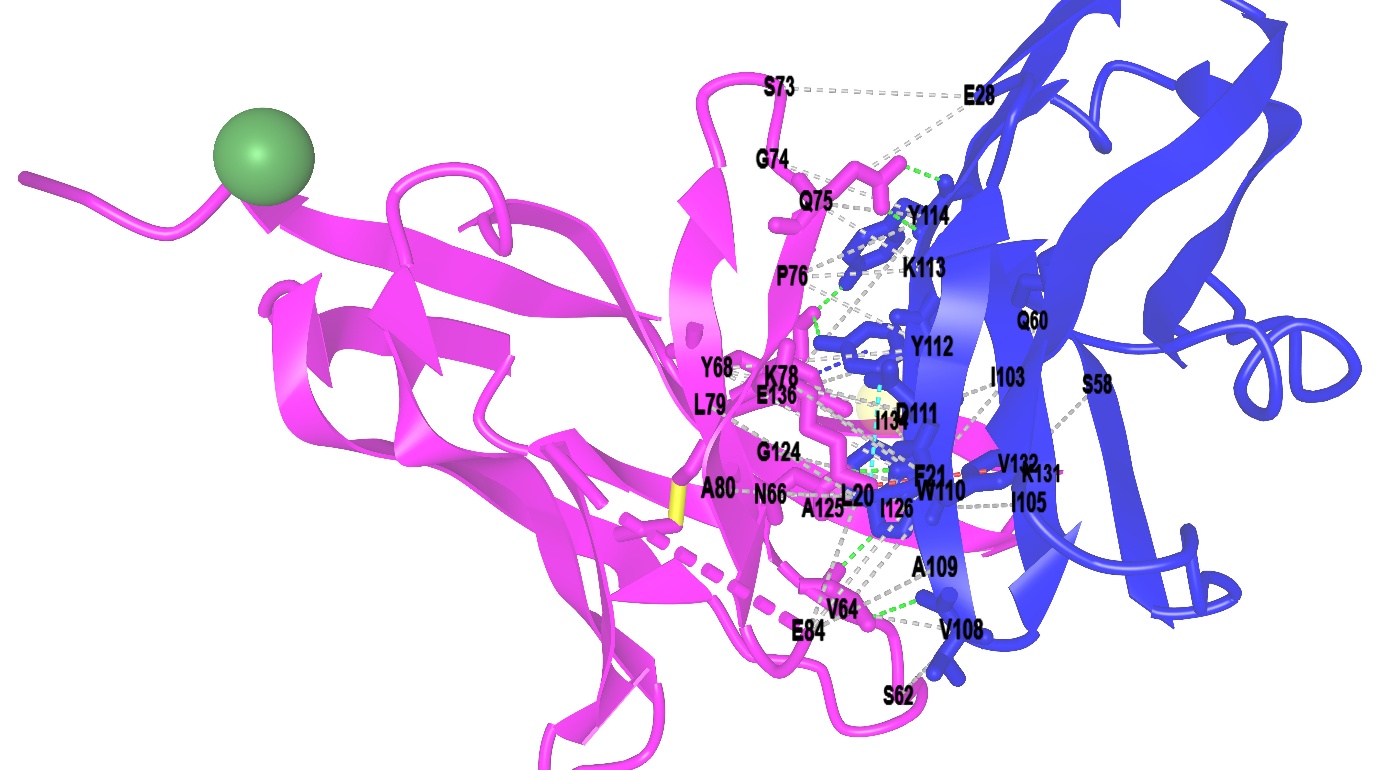


Designed Nanobody (Light blue) and PD-1(Orange) interactions:


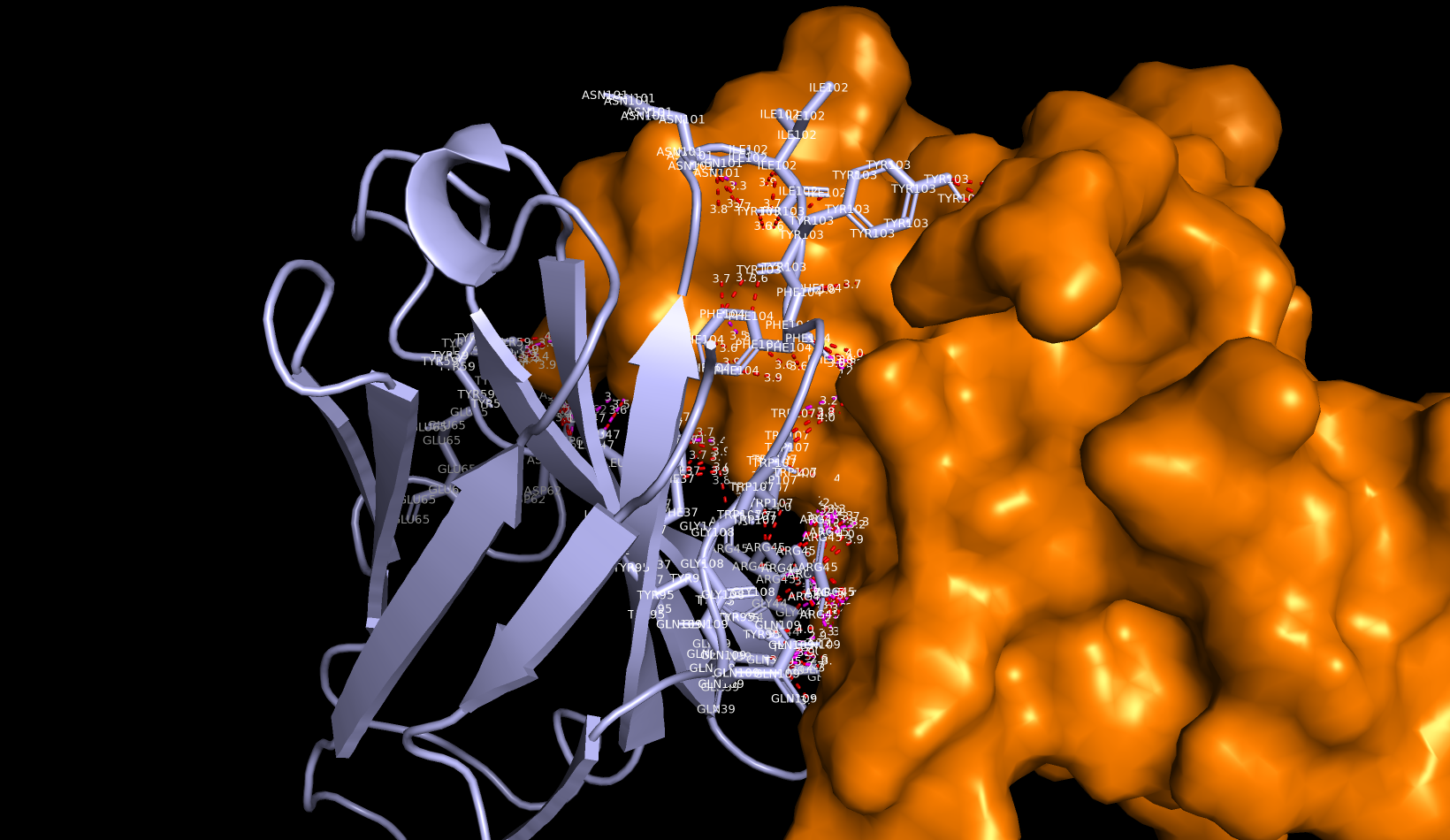


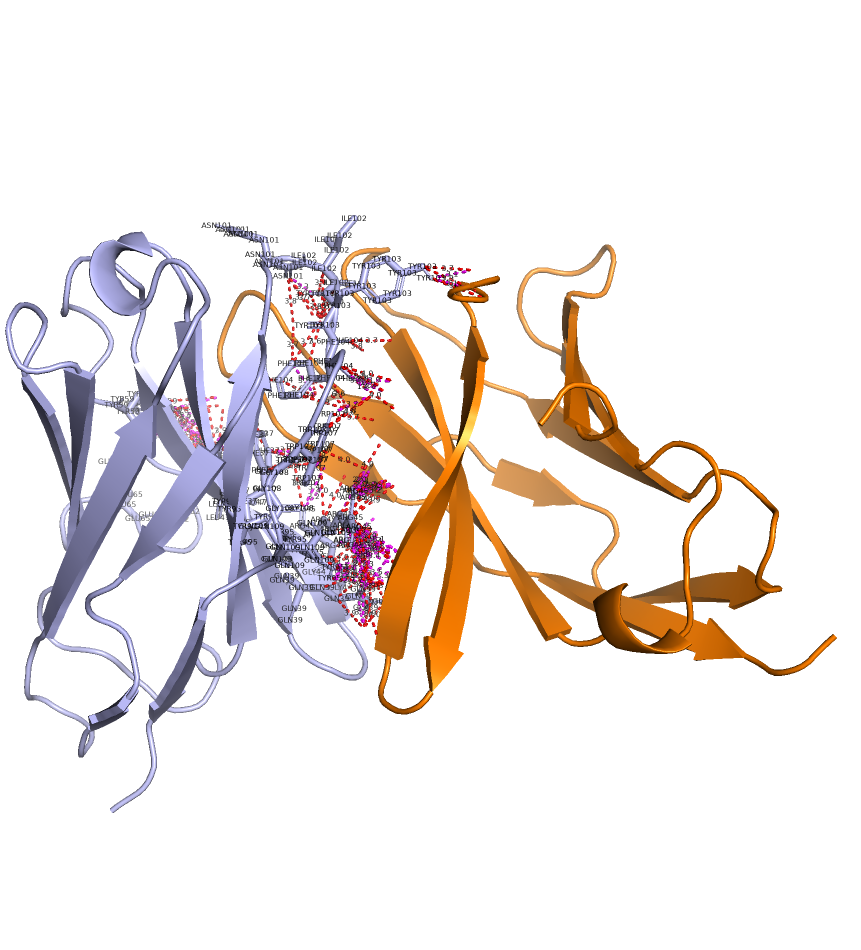


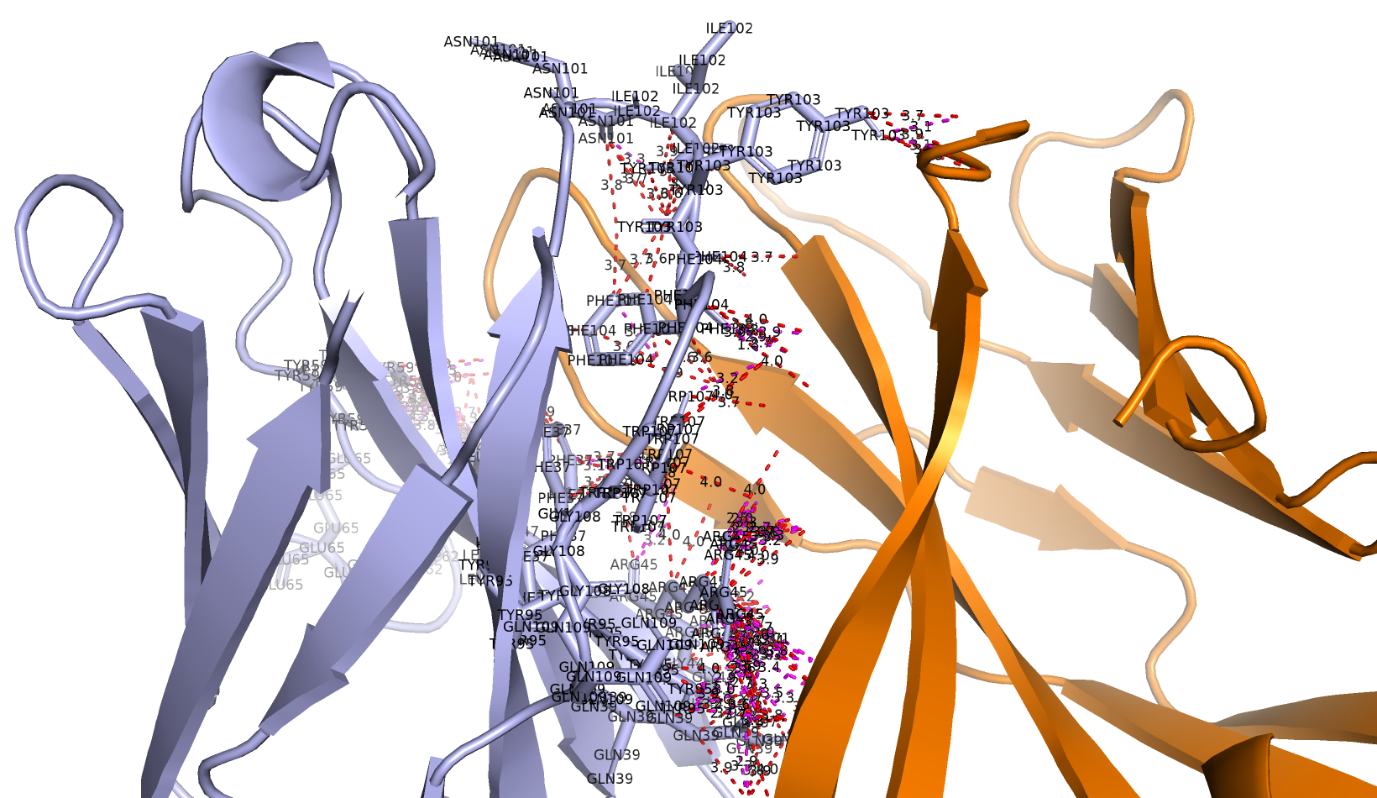

Supplement: Supplementary file 1 [file Supplementaryfile1.docx]
